# An unusual tropical endomyocardial fibrosis: a case report

**DOI:** 10.11604/pamj.2022.41.8.32886

**Published:** 2022-01-04

**Authors:** Hamid Jallal, Soufiane Belabbes, Ali Khatouri

**Affiliations:** 1 Department of Cardiology, Military Hospital of Instruction Omar Bongo Ondimba, Libreville, Gabon,; 2 Department of Cardiology, Military Hospital Avicenne, University Cadi Ayyad, Marrakech, Morocco

**Keywords:** Tropical endomyocardial fibrosis, cardiac calcification, eosinophilia, case report

## Abstract

Tropical endomyocardial fibrosis (TEF) is a rare condition that occurs primarily in tropical countries, leading to a severe heart failure with heart restrictive filling patterns. Eosinophilia appears to be a trigger leading to the development of the disease; thus, numerous etiologic factors accompanied by eosinophilia have been postulated, although none have been confirmed. The massively calcified form of TEF is exceptional and easily diagnosed by multimodal imaging; but it is a very rare condition with high surgical challenge. the best prevention remains the testing and treatment of parasitic infections frequently encountered in these countries.

## Introduction

Tropical endomyocardial fibrosis (TEF) also called Davies´s disease is the most common restrictive cardiomyopathy worldwide [1]. It is an enigmatic and idiopathic cardiomyopathy that occurs primarily in subtropical Africa, but other regions of the globe are affected as India, and South America, where it is a relatively frequent cause of congestive heart failure (HF). The disease affects mostly children and adolescents with ratio male/female of 1: 1, usually from low socioeconomic backgrounds. Its cause is unknown, but potential theories incriminate infection, autoimmunity, genetic predisposition, ethnicity, diet, climate, and poverty. The two most common consequences of this disease process are ventricular obliteration with restrictive diastolic filling and atrioventricular valve dysfunction caused by an extensive and progressive fibrosis of the ventricular endocardium. Death is caused either by congestive heart failure or malignant arrhythmias [2]. The goal of the surgical management was to increase the size and the compliance of the ventricular cavity and to preserve or restore atrioventricular valve competence, if necessary, it is advisable to perform surgery as early as possible before invasive endocardial fibrosis and significant calcification pairing which may limit or prevent the performance of surgery. Due to similitude of lesions observed in eosinophilic endomyocardial fibrosis (EMF), some authors have suggested that the two diseases have a common pathogenesis involving eosinophilic toxicity [3]; then eosinophil counts should be carefully assessed and hypereosinophilia treated as well as occasional parasitosis to avoid recurrence. The aim of this paper is to highlight how a neglected tropical cardiomyopathy can driving to the apical obstruction by a major calcification and limiting the surgical excision.

## Patient and observation

**Patient information:** a 60-year-old women, living in Libreville (Gabon), was referred for cardiologic evaluation with complaints of progressive exertional dyspnea and weakness for the last two years, she also had hacking cough and paroxysmal nocturnal dyspnea. There was no history of similar complaints in the family, she has never been declared hypertensive or diabetic and did not report drug reactions or allergies.

**Clinical findings:** on examination she had elevated jugular venous pressure, blood pressure 130/70mmHg; temperature 36.5°C; pulse 80/min; respiratory rate 18/min pulse rate and bilateral pitting pedal oedema. Cardiovascular examination revealed detected a systolic murmur in mitral area and rare bilateral crackles on pulmonary basis. Examination of the other systems was unremarkable.

**Timeline of current episode:** beginning of symptoms was on April 2019 and hospitalization in the cardiology department was on 4^th^ April 2021.

**Figure 1:**
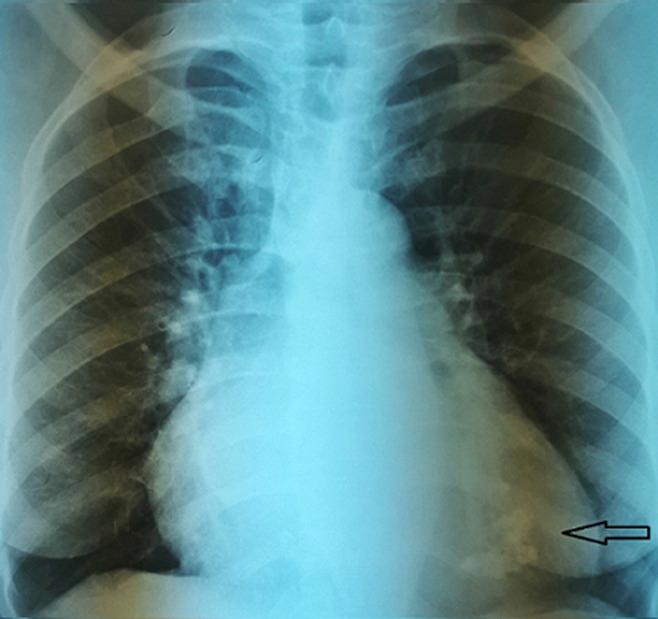
posterior- anterior chest X-ray finding a massively apical calcification of left ventricle

**Figure 2:**
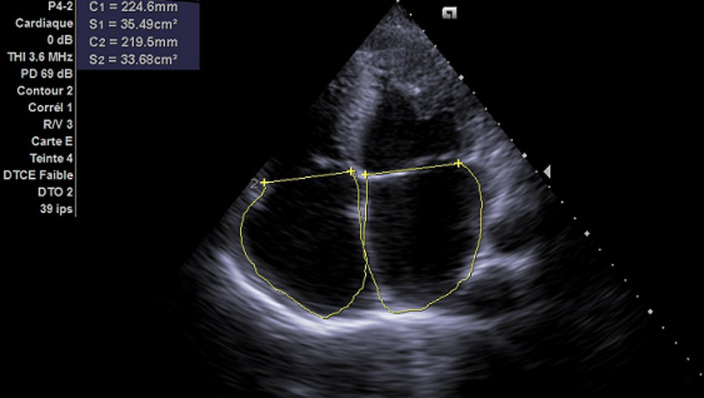
two-dimensional transthoracic echocardiography in apical 4-chamber view finding a noticeable reduction of left ventricular volume by a thrombotic materiel with calcification which also affects the mitral subvalvular apparatus

**Figure 3:**
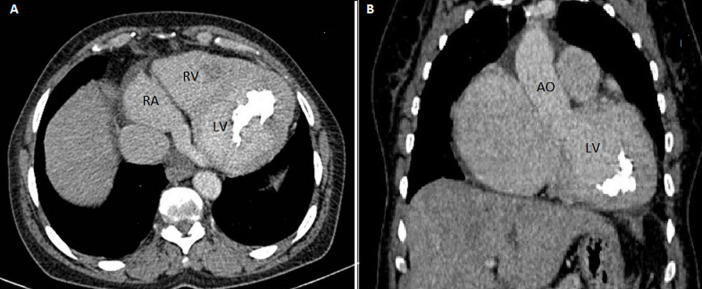
computed tomography (CT) cardiac scan; A) 4-chambre view; B) two chambre view shows a left ventricle apical obliteration due to a massive calcification which reached the posterior-medial papillary muscle

**Diagnostic assessment:** laboratory showed a very mild increase in C-reactive protein (2mg/dl), severe eosinophilia (3550/mL, 38%), neutropenia (1870/mL, 20%) and moderate increase in terminal probating natriuretic peptide (NT-Pro-Bnp 4825 pg/ml) whereas ultrasensitive troponin-I essay was negative (<15 ng/L) same as HIV serology. Electrocardiography revealed sinus rhythm, left ventricular hypertrophy and diffuse repolarization abnormalities. The chest X-ray showed mild cardiomegaly and marked calcification of the left ventricle (Figure 1). Echocardiography showed left ventricle obliteration of the apex by the thrombotic and calcified materiel which reached the posterior-medial papillary muscle (Figure 2), additional echo findings were bilateral atrial enlargement, mild dilatation, preserved ejection fraction (50%) but severe restrictive filling pattern of the left chamber (E/A ratio 3.3; E/E´ratio16) with at least mild mitral insufficiency and pulmonary hypertension (systolic pulmonary artery pressure 45mmHg). Cardiac computed tomography (CT) confirmed the presence of massified endomyocardial calcification in the apex which extent the posterior-medial papillary muscle (Figure 3 A,B). The set of clinical, laboratory, and imaging findings were suggestive of endomyocardial fibrosis. Test for serum total immunoglobulins type E was within the normal range, we excluded systemic malignancies and myeloproliferative disorder by thoracic -abdominal computed tomography and hematologic consultation. However, a stool examination revealed a salmonella infection treated by ciprofloxacin.

**Therapeutic intervention:** the patient was hospitalized and treated with conventional of heart failure based on loop diuretics, aldosterone receptor antagonist, angiotensin-converting enzyme inhibitor, betablocker and aspirin. Whereas the eosinophilia was treated with presumptive antiparasitic treatment based on ivermectin 6mg/day for 2 days and flubendazole 100mg single dose (repeat dose after 15 days), the eosinophil count was decreased (2200/ml). Unfortunately, the indication for surgery decortication was refused by the patient because of high operational risk.

**Follow-up and outcomes:** pedal oedema decreased, and she felt better. After two weeks, the patient was reviewed, with optimal treatment of congestion there was marked improvement of the dyspnea. It should be noted that after 1 month of presumptive antiparasitic treatment, the eosinophil count was decreased (2200/ml). At 3-month follow-up the patient was doing well, the eosinophil count was back to normal.

**Patient perspective:** during her hospitalization and after treatment, the patient was quite satisfied with the care she received and quite optimistic about the progress of her condition.

**Informed consent:** the patient consented to the publication of a case report containing her clinical data; because her case was rare, and she wanted to be helpful in the treatment of such a case in the future.

**Patient's consent:** informed consent was obtained from the patient for us to use the pictures.

## Discussion

Tropical endomyocardial fibrosis is a severe cardiac disease classified as a restrictive cardiomyopathy that occurs almost exclusively in patients living in tropical and subtropical regions. The hallmark of this disease is an extensive and progressive irregular fibrous thickening of the endocardium and subendocardial myocardium in the apex and inflow tract of one or both ventricles. The first necrotic stage called the inflammatory phase is characterized by eosinophilic infiltration of the myocardium associated with pancarditis and myocardial edema, vasculitis, and myocardial necrosis [4,5]. This phase is followed by formation of mural thrombi, often involving both ventricles, the ventricular outflow tracts, and the subvalvular regions resulting in thromboembolic complications; this process may lead to atrioventricular valvular incompetence [6]. The final stage occurred when the thrombus formed on denuded myocardium is replaced by fibrosis leading to a scarring of the chordae tendinea and endocardium resulting in a restrictive or dilated cardiomyopathy and progressive valvular incompetence [7] superimposed thrombosis and endocardial calcification is common in advanced cases. The TEF is inconsistently associated with eosinophilia [8] probably secondary to the eosinophilia of helminth infection [9] and supported by the high prevalence of parasitic infections in the tropics.

Thus, exact mechanisms of TEF remain unknown even if genetic predisposition [10] as well as malnutrition (protein and magnesium deficiency), toxic factors (cassava, cerium, thorium, serotonin, D-vitamin) were postulated [11]. Eosinophilia therefore appears to be a “trigger” which, with the association of certain environmental and/or genetic cofactors, will lead to the development of TEF. This combination of cofactors could explain the atypical geographical distribution of this disease. Calcified EMF is not an exceptional clinical form but the massively calcified obliteration of the apical region of the ventricular cavities in this condition is very rare. Cardiac CT scan is an accurate technique to evaluate the extent and anatomic distribution of endomyocardial calcification, while the echocardiography is limited due to the acoustic shadowing caused by the calcium. Surgery improves prognosis but remains poorly accessible in endemic areas [12], it consists in decortication of the fibrous endocardium and correction of atrioventricular valve regurgitation if needed but this type of surgery is technically challenging and limited by a high perioperative mortality [13] especially when it acts of massively calcified form as in our patient. The goal of the medical treatment is the to rapidly lower the eosinophil count and the fight against congestion with the conventional therapy of diastolic dysfunction.

## Conclusion

Tropical endomyocardial fibrosis continues to plague our tropical skies, the origin and pathophysiology of the disease is unknown. Echocardiography and, more recently, magnetic resonance imaging or cardiac CT scanner can demonstrate the typical lesions and constitute the most valuable techniques to confirm the diagnosis especially in calcified forms. Systematic screening strategies targeted at early diagnosis in specific high-risk groups would likely be very cost-effective and allow for more effective treatments before arriving at stages where surgery is unlikely.
